# Assessment of *Plasmodium falciparum* drug resistance associated molecular markers in Mandla, Madhya Pradesh, India

**DOI:** 10.1186/s12936-023-04817-7

**Published:** 2023-12-11

**Authors:** Akansha Singh, Mrigendra P. Singh, Nazia Anwar Ali, Rajan Poriya, Harsh Rajvanshi, Sekh Nisar, Sneha Bhandari, Ram S. Sahu, Himanshu Jayswar, Ashok K. Mishra, Aparup Das, Harpreet Kaur, Anup R. Anvikar, Ananias A Escalante, Altaf A. Lal, Praveen K. Bharti

**Affiliations:** 1 Indian Council of Medical Research-National Institute of Research in Tribal Health (ICMR-NIRTH), Jabalpur, Madhya Pradesh India; 2 Indian Council of Medical Research-National Institute of Malaria Research (ICMR-NIMR), New Delhi, India; 3 Malaria Elimination Demonstration Project, Mandla, Madhya Pradesh India; 4 Indian Council of Medical Research-National Institute of Research in Environment Health (ICMR-NIREH), Bhopal, Madhya Pradesh India; 5 Department of Health Services, Government of Madhya Pradesh, Mandla, Madhya Pradesh India; 6 Directorate of Health Services, Government of Madhya Pradesh, Bhopal, India; 7 Department of Health Research, Ministry of Health and Family Welfare, Indian Council of Medical Research, New Delhi, India; 8 Institute for Genomics and Evolutionary Medicine, Temple University, Philadelphia, PA USA; 9 Foundation for Disease Elimination and Control of India, Mumbai, Maharashtra India; 10 Global Health and Pharmaceuticals Inc., Atlanta, USA; 11 Present Address: University of Illinois, Urbana Champaign, Champaign, IL USA; 12 Present Address: Asia Pacific Leaders Malaria Alliance (APLMA), Singapore, Singapore; 13 Present Address: Department of Health and Family Welfare, NHM Raigarh, Chattisgarh, India

**Keywords:** *Plasmodium falciparum*, India, *Pfmdr1*, *Pfdhfr*, *Pfdhps*, *Pfk13* and artemisinin resistance

## Abstract

**Background:**

Resistance against artemisinin-based combination therapy is one of the challenges to malaria control and elimination globally. Mutations in different genes (*Pfdhfr*, *Pfdhps*, *Pfk-13* and *Pfmdr1*) confer resistance to artesunate and sulfadoxine–pyrimethamine (AS + SP) were analysed from Mandla district, Madhya Pradesh, to assess the effectiveness of the current treatment regimen against uncomplicated *Plasmodium falciparum.*

**Methods:**

Dried blood spots were collected during the active fever survey and mass screening and treatment activities as part of the Malaria Elimination Demonstration Project (MEDP) from 2019 to 2020. Isolated DNA samples were used to amplify the *Pfdhfr*, *Pfdhps*, *Pfk13* and *Pfmdr1* genes using nested PCR and sequenced for mutation analysis using the Sanger sequencing method.

**Results:**

A total of 393 samples were subjected to PCR amplification, sequencing and sequence analysis; 199, 215, 235, and 141 samples were successfully sequenced for *Pfdhfr*, *Pfdhps*, *Pfk13*, *Pfmdr1*, respectively. Analysis revealed that the 53.3% double mutation (C59R, S108N) in *Pfdhfr*, 89.3% single mutation (G437A) in *Pfdhps*, 13.5% single mutants (N86Y), and 51.1% synonymous mutations in *Pfmdr1* in the study area. Five different non-synonymous and two synonymous point mutations found in *Pfk13*, which were not associated to artemisinin resistance.

**Conclusion:**

The study has found that mutations linked to SP resistance are increasing in frequency, which may reduce the effectiveness of this drug as a future partner in artemisinin-based combinations. No evidence of mutations linked to artemisinin resistance in *Pfk13* was found, suggesting that parasites are sensitive to artemisinin derivatives in the study area. These findings are a baseline for routine molecular surveillance to proactively identify the emergence and spread of artemisinin-resistant parasites.

## Background

A global commitment and effort is underway to eliminate malaria, which is still a significant public health problem in several countries. In 2021, around 241 million malaria cases and 627,000 malaria deaths were observed globally [1]. India contributes around 83% of cases in the South East Asia Region (SEAR), of which 61% were *Plasmodium falciparum* and 39% *Plasmodium vivax* infections [1, 2]. Universal access to malaria diagnostics and treatment is part of the World Health Organization (WHO) strategic framework for countries progressing towards elimination. Over the last several decades, malaria elimination strategies deployed by many national programmes include case management using robust surveillance, artemisinin-based combination therapy (ACT) and vector control strategies using long-lasting insecticidal nets (LLINs) and indoor residual sprays (IRS). These efforts are constantly challenged by the emergence of anti-malarial drug resistance of parasites and insecticide resistance of vectors. *Plasmodium falciparum*, which causes severe forms of the disease, is more prone to develop resistance against the anti-malarials, as compared to other human infecting malaria parasites [3–5].

Historically, uncomplicated *P. falciparum* malaria was treated with chloroquine (CQ). After the development of resistance to CQ, sulfadoxine–pyrimethamine (SP) was deployed as the frontline anti-malarial drug. However, parasites resistant to SP emerged rapidly, leading to the change in drug policy to use ACT for the treatment of uncomplicated malaria. Artesunate + sulfadoxine–pyrimethamine (AS + SP), a combination of ACT, has been recommended by the Indian National Drug Policy for Malaria as frontline therapy for the treatment of uncomplicated *P. falciparum*, except in northeastern states of India, where artemether-lumefantrine (AL) was introduced [6] .

Resistance to sulfadoxine–pyrimethamine has been associated with Single Nucleotide Polymorphisms (SNPs) in the catalytic site of the enzymes dihydropteroate synthase (*Pfdhps*) and dihydrofolate reductase (*Pfdhfr*), respectively. Point mutations at codons 16, 51, 59, 108, and 164 of *Pfdhfr* confer resistance to pyrimethamine, whereas mutations at 436, 437, 540, 580 and 613 of *pfdhps* prevent the activity of sulfadoxine [3, 7–12]. Polymorphism in the propeller domain of *Pfkelch13* (*Pfk13*) has been found to be associated with artemisinin resistance [13, 14]. SNPs at codons 493, 539, 543, and 580 of *Pfk13* gene are responsible for artemisinin resistance, whereas P553L has been associated with delayed parasite clearance in Southeast Asia [15].

Furthermore, most anti-malarials metabolise through ABC transporter and SNPs at codons N86Y and Y184F of *Pfmdr1* confer resistance against partner drugs, such as mefloquine and lumefantrine thus limiting the therapeutic activity of artemisinin-based combinations [16]. Since these mutations are linked to anti-malarial resistance, tracking them in the natural parasite population is an effective way to monitor for potential problems with anti-malarial drug efficacy, even before there are clinical reports [17]. Baseline information about polymorphisms in marker genes associated with anti-malarial drug resistance will be helpful to predict the emergence of resistance among the parasite population in a given geographical region.

ACT was implemented starting in 2010 in Mandla district for the treatment of uncomplicated *P. falciparum* malaria. Therefore, the present study was conducted to assess the point mutations in marker genes *Pfdhfr*, *Pfdhps*, *Pfk13*, and *Pfmdr1* associated with anti-malarial drug resistance among *P. falciparum* samples collected from Mandla, Madhya Pradesh, as a part of the Malaria Elimination Demonstration Project (MEDP).

## Methods

### Study area, population, and sample collection

This study was part of the Malaria Elimination Demonstration Project, which was a first-of-its-kind public-private-partnership between the Indian Council of Medical Research (ICMR) through the National Institute for Research in Tribal Health (NIRTH) Jabalpur, Government of Madhya Pradesh (GoMP), and the Foundation for Disease Elimination and Control of India (established by Sun Pharmaceutical Industries Ltd. as a not-for-profit entity) [18].

**Fig. 1:**
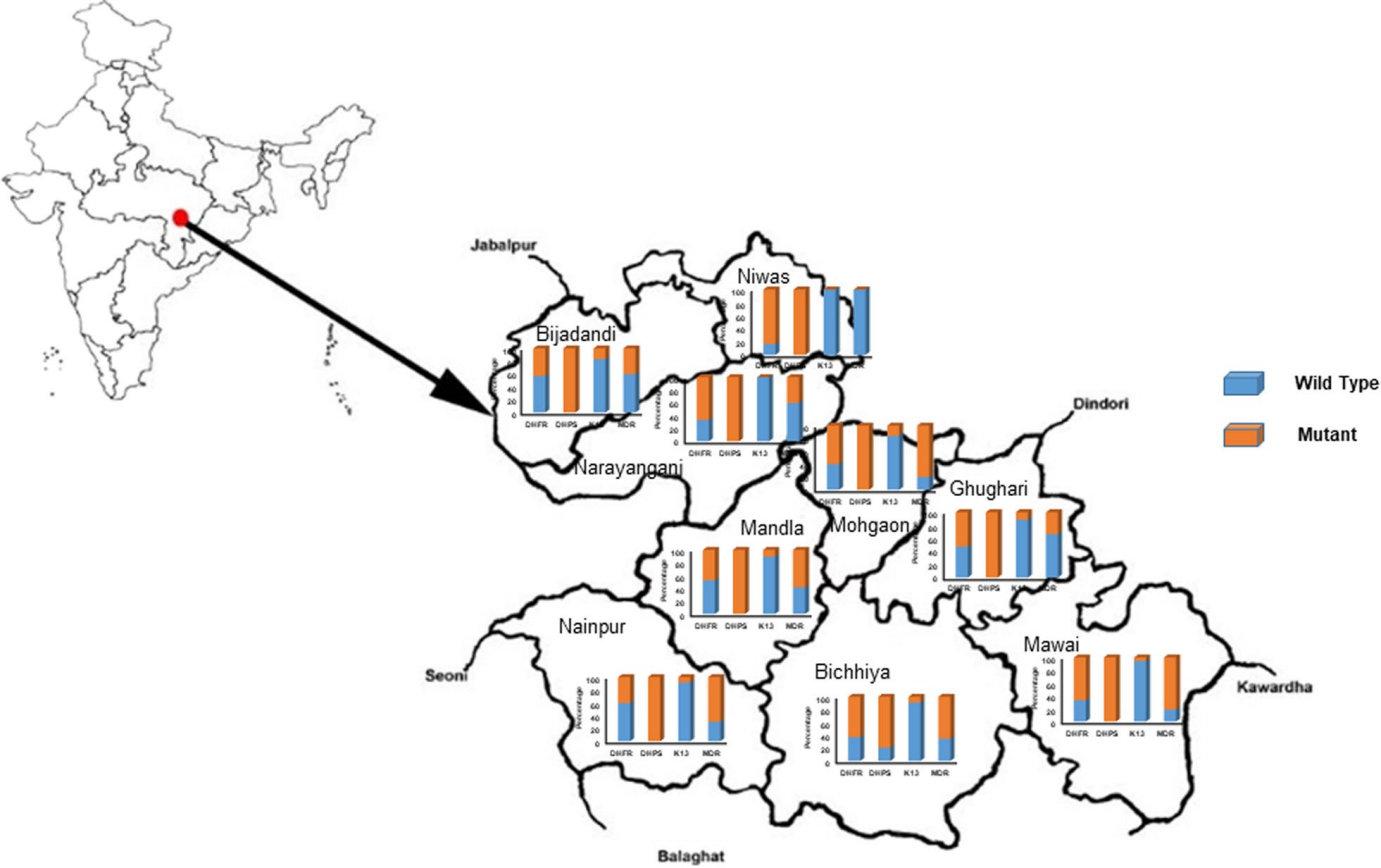
Map of India showing Mandla district in Madhya Pradesh, the study site divided in nine different block with different level of mutation in anti-malarial resistance markers

This study was carried out in Mandla district, Madhya Pradesh (between geo-coordinates 22° 02′ and 23° 22′ N and 80° 18′ and 81° 50′ E), which is mainly inhabited by Scheduled Tribes (59%) [19]. The district has an area of 8771 km^2^ divided into nine development blocks (Fig. 1) and 1233 villages; the total population is 1,140,765 population [18]. Dried blood spots were collected from finger pricks as described previously during the active door-to-door fever survey [20] and Mass Screening and Treatment (MSaT) strategies [21] during 2019–2020. The detailed methodology of sample collections and results of malaria diagnosis using rapid diagnostic tests, microscopic smear examinations, and Polymerase Chain Reaction (PCR) findings were reported elsewhere [20–22].

### Genomic DNA extraction and parasite genotyping

Molecular analysis was done from 393 *P. falciparum*-positive dried blood spot samples during 2019–2020. Genomic DNA was isolated from the dried blood spots using the Chelex method, as described earlier [21, 22]. The presence of *Plasmodium* species was determined using species-specific nested PCR by targeting 18Sr RNA gene, a technique reported elsewhere [21, 22].

A two-step nested PCR was performed for four resistance-conferring genes (*Pfdhfr*, *Pfdhps*, *Pfk13* and *Pfmdr1*). The *Pfdhfr* gene (542 bp, 15–170 aa) was amplified to analyse mutations associated with pyrimethamine resistance (in codons 16, 50, 51, 59, 108, and 164). The *Pfdhps* gene (735 bp, 425–650 aa) was analysed to identify mutations associated with sulfadoxine resistance (codons 436, 437, 540, 581, 613). The propeller region of the *Pfk13* gene (849 bp, 427–709 aa) was amplified to identify validated mutations (codons 446, 458, 476, 493, 539, 543, 553, 561, and 580) and candidate associated mutations (codons 441, 449, 469, 481, 537, 538, 568, 574, 672, 673, and 675) for artemisinin resistance. *Pfmdr1* gene (856 bp, 47–332 aa) was amplified to identify mutations atcodons 86, 184 associated with antimalarial drug resistance. The details of PCR primers and cycling conditions are given in Table 1. In brief, PCR was performed in a volume of 25 µL with 0.2 U of Taq polymerase enzyme (Invitrogen, life technologies), 0.2 mM each dNTP, 1 µM each primer and 1.5 mM MgCl_2_.

**Table 1:** Primer sequence and PCR condition used for amplification of *P. falciparum* drug resistance genes

Gene	Primer name	Primer sequence	PCR product length (bp)	Denaturation	Annealing	Elongation	No of cycles
*Pfmdr1* primary	MDR1	AGAGTACCGCTGAATTATTTAG	1472	94 °C, 1 min	53 °C, 1 min	72 °C, 1 min	35
	MDR2	TTCATTTGATGTCATAGAATTCG					
*Pfmdr1* nested	MDR3	ATGTTTACCTGCACAACATAGAA	856	94 °C, 1 min	52 °C, 1 min	72 °C, 1 min	30
	MDR4	CATAAACATACTAATAAGTACACC					
*Pfdhfr* primary	PF1	TTTATATTTTCTCCTTTTTA	718	94 °C, 1 min	45 °C, 45 s	72 °C, 1 min	35
	PR1	CATTTTATTATTCGTTTTCT					
*Pfdhfr* nested	PF1	TTTATATTTTCTCCTTTTTA	648	94 °C, 1 min	45 °C, 45 s	72 °C, 1 min	30
	NR2	ACAGAAATAATTTGATACTCA					
*Pfdhfs* primary	F1	CCATTCCTCATGTGTATACAACAC	1167	94 °C, 1 min	55 °C, 1 min.	72 °C, 1 min. 30 s	35
	R1	CTTGGTCTATTTTTGTTAAAACATCC					
*Pfdhfs* nested	F2	TGGAATATTAAATGTTAATTATGA	735	94 °C, 1 min	50 °C, 45 s	72 °C, 1 min	30
	R2	TTTTCATTTTGTTGTTCATCATGT					
*PfK 13*	K13_F	GCCAAGCTGCCATTCATTTG	849	94 °C, 45 s	60 °C, 1 min	72 °C, 1 min 30 s	35
	K13_R	GCCTTGTTGAAAGAAGCAGA					

*Pfmdr1*: *Plasmodium falciparum* multi drug resistance gene1; *Pfdhfr*: *Plasmodium falciparum* dihydrofolate reductase, *Pfdhps*: *Plasmodium falciparum* dihydropteroate synthase; *Pfk13*: *Plasmodium falciparum* kelch13, °C: temperature in degree Celsius; min: minute; s: seconds; bp: base pair; n = number; %= percentage

### Nucleotide sequencing

The PCR amplicons were purified using exonuclease I and shrimp alkaline phosphatase, following the manufacturer’s instructions. The purified product was used with the ABI Big dye Terminator Ready Reaction Kit Version 3.1 for DNA sequencing. The sequencing PCR was performed in a volume of 10 µL with 0.5 µL of Terminator Ready Reaction Mix (TRR), 3.2 pmol of gene specific forward and reverse primer (both directions) and sequencing buffer. Cycling conditions for the sequencing PCR include 25 cycles of denaturation at 96 °C for 10 s, annealing at 50 °C for 5 s and extension at 60 °C for 4 min. Sequencing was performed on a 3130XL genetic analyser (Applied Biosystems, USA). Sequencing results were analysed by V5.4 software (Applied Biosystems, USA), and contigs were prepared using Bioedit sequence alignment editor version 7.2.3.

### Statistical analysis

Data was entered in Microsoft Excel 2013 Worksheet. Qualitative (Categorical) variables were coded numerically, and frequency with percentage distribution was tabulated. Pearson’s Chi-square or Fisher’s exact test was applied for appropriate statistical comparison of independent proportions. Bivariable logistic regression analysis was performed to estimate the association of independent factors such as endemicity, age group, symptoms, and year with *Pfdhfr*, *Pfdhps*, *Pfk13*, and *Pfmdr1* gene mutations. All the statistical analysis has been performed using R version 4.2.2 for Windows (R Foundation for Statistical Computing, Vienna, Austria).

## Results

A total of 335 out of 17,405 during the active fever survey and 221 out of 24,357 samples during the MSaT were *P. falciparum* positive by PCR. A total of 393 out of 556 *P. falciparum* positive samples were available in sufficient quantity and used for molecular genotyping of *Pfdhfr*, *Pfdhps*, *Pfk-13* and *Pfmdr1* genes using the Sanger sequencing method and good read from both the direction were used for the sequence analysis. Mutations were identified with using 3D7 as reference strain.

### Analysis of *pfdhfr* and *pfdhps* mutations

**Fig. 2:**
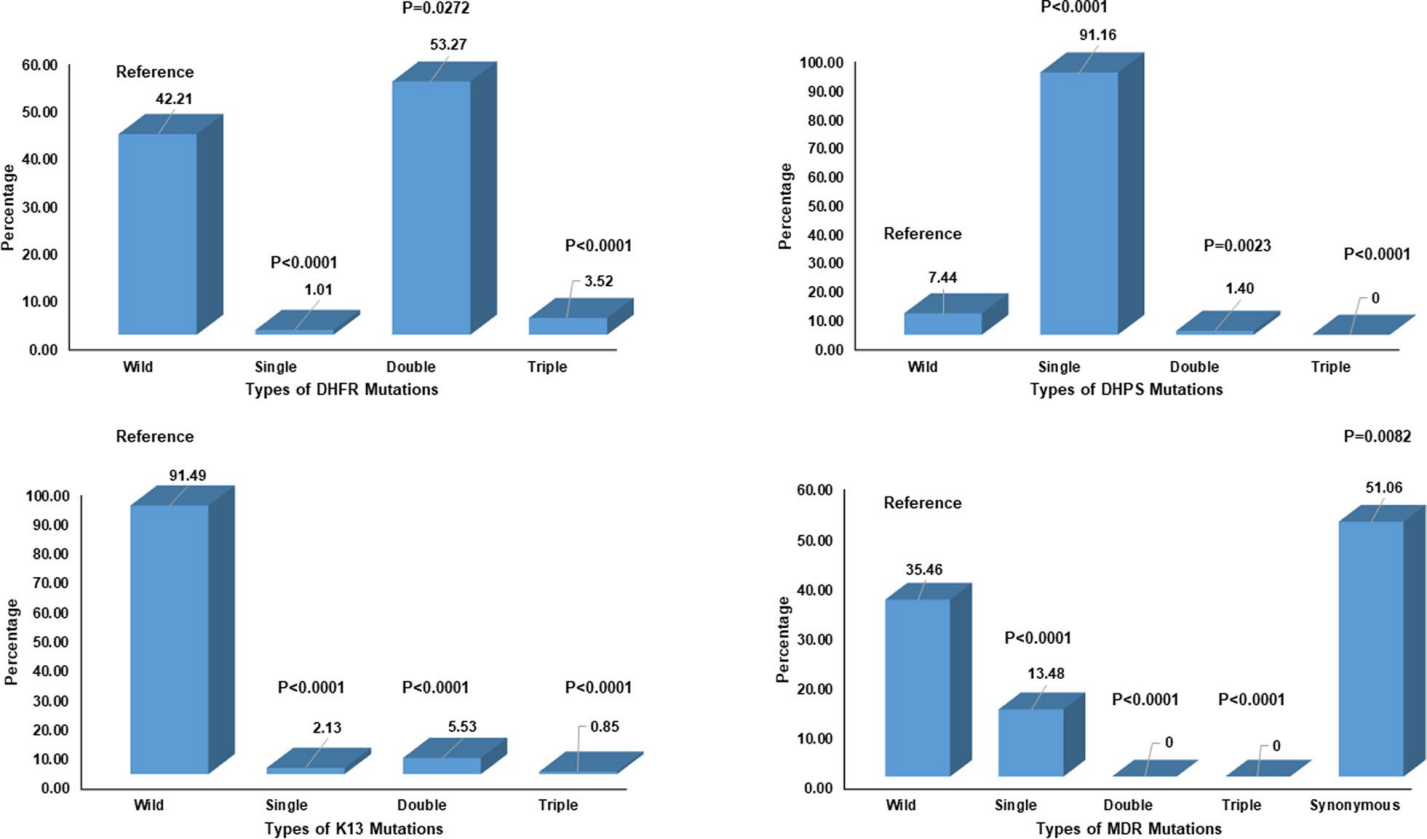
Mutation in *P. falciparum* genes (*Pfdhfr*, *Pfdhps*, *pfk13* and *Pfmdr1*) that confer the resistance to anti-malarials

A total 199 *P. falciparum* samples were successfully analysed for both *Pfdhfr* and *Pfdhps* genes. Out of the five *Pfdhfr* mutations (A16V, N51I, C59R, S108N/T, and I164l) conferring pyrimethamine resistance, only three mutations at codons N51I, C59R, and S108N were found as single, double or triple mutations (Table 2). In addition to the above five functional mutations, we have found a novel mutation at L46S in the triple mutant combination. Overall, 58% of parasite isolates were found to harbour mutant genotypes, while only 42% had wild-type (sensitive) genotypes. Majority of them were double mutants (53.3%) at codon C59R and S108N followed by triple mutants (3.5%) and 1.0% single mutants (Fig. 2). Four mutant haplotypes were determined in the *Pfdhfr* gene. More (62%, 84/135) samples were found with resistant mutants in 2020 compared to 2019 (48%, 31/64). No significant difference in the prevalence of mutant genotype was observed among the symptomatic vs. asymptomatic cases, adult vs. children, and in different malaria endemic areas (Table 3).

**Table 2:** Different haplotype of *Pfdhfr* gene and their association with *Pfdhps* haplotypes (NIL: dhps gene didn’t successfully analysed and WILD TYPE: no mutations in the analysed samples)

*Pfdhfr*	*Pfdhps*							Total
	NIL	G437A	G437A, I514M	P444S, R569C	S436A	S436A, K540E	Wild type	
C59R, S108N								
N	16	78	0	1	0	1	10	106
%	15.10%	73.60%	0.00%	0.90%	0.00%	0.90%	9.40%	100.00%
L46S, C59R, S108N								
N	0	0	0	0	0	0	1	1
%	0.00%	0.00%	0.00%	0.00%	0.00%	0.00%	100.00%	100.00%
N51I, C59R, S108N								
N	2	0	0	0	4	0	0	6
%	33.30%	0.00%	0.00%	0.00%	66.70%	0.00%	0.00%	100.00%
S108N								
N	2	0	0	0	0	0	0	2
%	100.00%	0.00%	0.00%	0.00%	0.00%	0.00%	0.00%	100.00%
Wild type								
N	11	70	0	0	0	0	3	84
%	13.10%	83.30%	0.00%	0.00%	0.00%	0.00%	3.60%	100.00%
Total								
N	31	148	0	1	4	1	14	199
%	15.58%	74.37	0.00%	0.50%	2.01%	0.50%	7.04%	100.00%

**Table 3:** Logistic regression analysis of the various factors associated with the different anti-malarial markers from the study sites

Factors	*Pfdhfr*		*Pfdhps*		*Pfmdr1*	
	n/d (%)	OR (95% CI)	n/d (%)	OR (95% CI)	n/d (%)	OR (95% CI)
Endemicity						
Low	12/19 (63.16)	Reference	17/17 (100)	Reference	4/11 (36.36)	Reference
Moderate	30/65 (46.15)	0.50 (0.17–1.43)	77/77 (100)	Empty	27/47 (57.45)	2.26 (0.61–9.18)
High	73/115 (63.48)	1.10 (0.37–2.77)	105/121 (86.78)	Omitted	60/83 (72.29)	4.56 (1.22–17.07)*
Age group						
Adults	90/158 (56.96)	Reference	161/170 (94.71)	Reference	74/117 (63.25)	Reference
Children	25/41 (60.98)	1.18 (0.58–2.38)	38/45 (84.44)	0.30 (0.11–0.87)*	17/24 (70.83)	1.41 (0.54–3.67)
Symptoms						
Symptomatic	96/166 (57.83)	Reference	162/177 (91.53)	Reference	69/105 (65.71)	Reference
Asymptomatic	19/33 (57.58)	0.98 (0.46–2.11)	37/38 (97.37)	3.43 (0.44–26.76)	22/36 (61.11)	0.82 (0.37–1.79)
Year						
2019	31/64 (48.44)	Reference	70/74 (94.59)	Reference	43/68 (63.24)	Reference
2020	84/135 (62.22)	1.75 (0.96–3.20)	129/141 (91.49)	0.61 (0.19–1.98)	48/73 (65.75)	1.12 (0.56–2.23)

*p < 0.05

Out of the five *Pfdhps* mutations (S436A, A437G, K540E, A581G, and A613T) known to be linked to sulphadoxine resistance, three codon S436A, A437G and K540E were found as single or double mutants (Table 2). In addition to the above functional mutations, one sample showed mutation at P444S, R569C and I514M. The wild type was present in only 16/215 (7.5%) samples. The single mutant G437A was detected in 192/215 (89.3%) samples, followed by the S436A mutation in 4/215 (1.8%). The study found double mutants in each of three different combinations (S436A, K540E; G437A, I514M; P444S, R569C) (Fig. 2; Table 2).

When the data of mutations of the two genes (*Pfdhfr* and *Pfdhps*) was analysed, we found that only 1% was wild type. A quadruple mutant of *Pfdhfr* (N51I, C59R, S108N) and *Pfdhps* (S436A) was found in only four cases. However, the triple mutant *Pfdhfr* (C59R, S108N) and *Pfdhps* (G437A) were found in 78/106 (73.6%) samples (Table 2). The study has not found any resistant associated mutations for *Pfdhfr* at codon 164 and for *Pfdhps* at codons 581 or 613. The six samples with triple mutant *Pfdhf*r N51I, C59R, S108N were distributed throughout the district (two cases from low endemic areas, three from moderate and one from high) in 2020. Similarly, four *pfdhps* mutations at codon S436A were distributed throughout the districts (one case from low endemic areas, two from moderate and one from high) in 2020. Overall, there was no significant difference in the distribution of *Pfdhfr* and *Pfdhps* mutants observed over the 2-year study period (Fig. 1).

### Analysis of *Pfk-13* mutations

The propeller region of the *Pfk-13* gene was successfully sequenced and analysed from 235 *P. falciparum* samples. The study revealed that 91% of the samples harboured wild-type mutation, while 9% of the sample showed 2% single (F506S, I634M, M579T, N657H), 6% double (M579T-N657H and R597G-T677S) and 1% triple (M579T-N599H-N657H and M579T-N657H-K658E) mutations (Fig. 2). None of these mutations have been shown to be responsible (validated codons 446, 458, 476, 493, 539, 543, 553, 561, and 580; associated codons 441, 449, 469, 481, 537, 538, 568, 574, 672, 673, and 675) for conferring artemisinin resistance. A total of 11 different mutant haplotypes were found in this study in a smaller number of samples, and no association with the *Pfmdr1* genotype.

### Analysis of *Pfmdr1* mutations

The *Pfmdr1* gene was analysed from 141 samples, and mutation at N86Y was found in 13.5% samples and did not find any mutations at codon Y184F (Fig. 2). The study revealed that 51% of samples had synonymous mutations at different codons (182, 183, 215, 231 and 271).

The study has also analysed various independent factors associated with antimalarial drug resistance markers, such as malaria endemicity, age group, symptoms, and year of sample collection. The analysis revealed a significantly higher number of mutations in *Pfmdr1* in high endemic areas, *Pfk13* in the asymptomatic group, and *Pfdhps* in children under 14 years of age (Table 3).

## Discussion

Since 2010, ACT has been used in India to treat uncomplicated *P. falciparum* malaria after significant resistance was found against CQ and SP [12, 23–33]. Due to the emergence and spread of anti-malarial drug-resistant parasites, monitoring of molecular markers has become an essential component of malaria control strategies. The present study investigated the mutations in the *Pfdhfr*, *Pfdhps*, *Pfk13* and *Pfmdr1* genes of *P. falciparum* samples collected as part of MEDP.

Mutations in *Pfdhfr* and *Pfdhps* genes associated with SP resistance have been reported in India and other countries, and parasites harbouring quintuple mutations of *Pfdhps* (437G and 540E) and *Pfdhfr* (51I, 59R, and 108N) genes are known to be resistant to SP [34, 35]. It has been documented that single point mutation in these two genes signals early signs of the improper action of the drugs, while the double mutations may indicate a decreased parasite sensitivity to the drug, and multiple mutations (triple or more) raise concerns for drug failures [36]. In the present study, the triple mutations in the *Pfdhfr* (C59R, S108N) and *Pfdhps* (G437A) genes were found in 73.6% of samples, which indicates circulation of possible resistant forms of *P. falciparum* parasites in the study area. Similar triple mutation-bearing parasites have been reported in central India [9, 26, 27, 37–39]. However, studies conducted in the eastern (Odisha, West Bengal) and North Eastern states have revealed high levels of (quadruple and quintuple) mutations that are associated with drug failures [11, 24, 26, 40–43].

In the present study, only four samples out of 235 were found to have quadruple mutants of *Pfdhfr* (N51I, C59R, S108N) and *Pfdhps* (S436A). Molecular surveillance should be continued in the study area to monitor the emergence and spread of SP resistance as low level of quadruple mutants have already been observed.

With India’s proximity to Cambodia and the Greater Mekong Subregion (GMS), where artemisinin (ART) resistant forms for parasites have emerged, studies of molecular surveillance to document mutations in the *k13 propeller* genes are essential [13, 15, 44]. Although 108 non-synonymous mutations from the different geographic regions of the world have been identified [45, 46] only nine-point mutations i.e., codons 446, 458, 476, 493, 539, 543, 553, 561, and 580) are known to confer ART resistance [47]. Other 11 different point mutations at codons 441, 449, 469, 481, 537, 538, 568, 574, 672, 673, and 675 have been associated with late parasite clearance. A limited number of validated mutations (F446I, R539T, R561H) and resistance-associated mutations (A481V, N672S, A675V) have been previously reported from West Bengal and the Northeastern States of India [48–51]. However, eight other mutations have been found in India (K189T, G533A, S549Y, A578S, M579T, G625R, N657H, D702N), which are not associated with ART resistance [48–52]. In the present study, out of eight documented mutations (F506S, M579T, R597G, N599H, I634M, N657H, K658E, T677S), mutations M579T and N657H have been identified for the first time in India. These observations indicate that, unlike SP, ART is not under selection pressure at this time.

Mutation in *Pfmdr1* N86Y is known to contribute to multi-drug resistance. In this study, 13.5% of samples were found to bear N86Y mutation, and 51% had synonymous mutations. The study has also revealed that synonymous mutations were neither at codon 86 nor at 184, which are crucial for conferring drug resistance. Studies have shown that the prevalence of N86Y mutation varies across the country and the South East Asia region depending upon the drug pressure and transmission intensity [16, 28, 32, 33, 39, 53]. It has been shown that N86Y mutation has a positive modulation effect when present in combination with other drug-resistant mutations. These observations indicate that the dynamic nature of evolving parasite populations exposed to different antimalarial drugs may influence the emergence of drug resistance. The triple mutant *Pfdhf*r N51I, C59R, S108N were distributed throughout the district in 2020. This could be due to several reasons for the higher number of mutations in the year 2020 as compared to 2019. MEDP started the active fever cases screening and treatment in the month of September 2017 and continued till 2021 to eliminate the indigenous malaria cases from the study area. The project also carried out MSaT strategies to resolve the malaria hotspots and asymptomatic cases during 2018 and 2019, which could have put drug pressure on the parasite resulting in the emergence of mutationsThe present study provides the status of molecular markers associated with the *P. falciparum* drug resistance. However, the therapeutic efficacy and in-vitro assessment were not part of the study. Such studies should be carried out in future if there are reports of delays/failures of ACT.

## Conclusion

The study found no mutations linked to ART resistance in the *Pfk13*. However, the finding of triple mutations in *Pfdhfr* and *Pfdhps* could present a problem for the use of AS + SP for the treatment of uncomplicated *P. falciparum* malaria in the study areas. Therefore, there is a need for continued molecular surveillance to quantify the presence of mutation-bearing parasites. Molecular surveillance, together with *in-vivo* efficacy and therapeutic efficacy studies using appropriate epidemiologic designs, would guide policymakers to make appropriate decisions for making any changes in the treatment regimen.

## Data Availability

We have reported all the findings in this manuscript. The hardcopy data is stored at MEDP Office in Jabalpur, Madhya Pradesh, and Indian Council of Medical Research-National Institute of Research in Tribal Health (ICMR-NIRTH), Jabalpur, Madhya Pradesh. Softcopy data is available on the project server of MEDP hosted by Microsoft Azure. Molecular data is already submitted to the NCBI database. If anyone wants to review or use the data, they should contact: Dr. Praveen Kumar Bharti.Scientist E, ICMR—National Institute of Malaria Research, New Delhi, India and Dr. Altaf A. Lal. Project Director—Malaria Elimination Demonstration Project, Mandla. Foundation for Disease Elimination and Control of India, Mumbai, India 482003. E-mail: altaf.lal@sunpharma.com, altaf.lal@gmail.com.

## References

[1] WHO. World malaria report 2022. Geneva: World Health Organization. 2022. https://www.who.int/publications-detail-redirect/9789240064898.

[2] Malaria situation in India national vector borne disease control programme (NVBDCP). https://nvbdcp.gov.in/index4php?lang=1&level=0&linkid=564&lid=3867.

[3] Uwimana A, Umulisa N, Venkatesan M, Svigel SS, Zhou Z, Munyaneza T (2021). Association of *Plasmodium falciparum* kelch13 R561H genotypes with delayed parasite clearance in Rwanda: an open-label, single-arm, multicentre, therapeutic efficacy study. Lancet Infect Dis.

[4] McCollum AM, Schneider KA, Griffing SM, Zhou Z, Kariuki S, ter Kuile F (2012). Differences in selective pressure on dhps and dhfr drug resistant mutations in western Kenya. Malar J.

[5] Escalante AA, Smith DL, Kim Y (2009). The dynamics of mutations associated with antimalarial drug resistance in *Plasmodium falciparum*. Trends Parasitol.

[6] National drug policy on malaria. https://www.nvbdcp.gov.in/Doc/National-Drug-Policy2013.pdf.

[7] Plowe CV, Kublin JG, Doumbo OK (1998). *P. falciparum* dihydrofolate reductase and dihydropteroate synthase mutations: epidemiology and role in clinical resistance to antifolates. Drug Resist Updat.

[8] Wicht KJ, Mok S, Fidock DA (2020). Molecular mechanisms of drug resistance in *Plasmodium falciparum* malaria. Annu Rev Microbiol.

[9] Patel P, Bharti PK, Bansal D, Ali NA, Raman RK, Mohapatra PK (2017). Prevalence of mutations linked to antimalarial resistance in *Plasmodium falciparum* from Chhattisgarh, Central India: a malaria elimination point of view. Sci Rep.

[10] Ahmed A, Bararia D, Vinayak S, Yameen M, Biswas S, Dev V (2004). *Plasmodium falciparum* isolates in India exhibit a progressive increase in mutations associated with sulfadoxine–pyrimethamine resistance. Antimicrob Agents Chemother.

[11] Chaturvedi R, Chhibber-Goel J, Verma I, Gopinathan S, Parvez S, Sharma A (2021). Geographical spread and structural basis of sulfadoxine–pyrimethamine drug-resistant malaria parasites. Int J Parasitol.

[12] Alam MT, Vinayak S, Congpuong K, Wongsrichanalai C, Satimai W, Slutsker L (2011). Tracking origins and spread of sulfadoxine-resistant *Plasmodium falciparum* dhps alleles in Thailand. Antimicrob Agents Chemother.

[13] Dondorp AM, Nosten F, Yi P, Das D, Phyo AP, Tarning J (2009). Artemisinin resistance in *Plasmodium falciparum* malaria. N Engl J Med.

[14] Imwong M, Suwannasin K, Kunasol C, Sutawong K, Mayxay M, Rekol H (2017). The spread of artemisinin-resistant *Plasmodium falciparum* in the greater Mekong subregion: a molecular epidemiology observational study. Lancet Infect Dis.

[15] Ariey F, Witkowski B, Amaratunga C, Beghain J, Langlois A-C, Khim N (2014). A molecular marker of artemisinin-resistant *Plasmodium falciparum* malaria. Nature.

[16] Pickard AL, Wongsrichanalai C, Purfield A, Kamwendo D, Emery K, Zalewski C (2003). Resistance to antimalarials in Southeast Asia and genetic polymorphisms in pfmdr1. Antimicrob Agents Chemother.

[17] Escalante AA, Ferreira MU, Vinetz JM, Volkman SK, Cui L, Gamboa D (2015). Malaria molecular epidemiology: lessons from the international centers of excellence for malaria research network. Am J Trop Med Hyg.

[18] Rajvanshi H, Bharti PK, Nisar S, Jain Y, Jayswar H, Mishra AK (2020). Study design and operational framework for a community-based malaria elimination demonstration project (MEDP) in 1233 villages of district Mandla, Madhya Pradesh. Malar J.

[19] Sharma RK, Rajvanshi H, Bharti PK, Nisar S, Jayswar H, Mishra AK (2021). Socio-economic determinants of malaria in tribal dominated Mandla district enrolled in malaria elimination demonstration project in Madhya Pradesh. Malar J.

[20] Bharti PK, Rajvanshi H, Nisar S, Jayswar H, Saha KB, Shukla MM (2020). Demonstration of indigenous malaria elimination through track-test-treat-track (T4) strategy in a malaria elimination demonstration project in Mandla, Madhya Pradesh. Malar J.

[21] Singh A, Rajvanshi H, Singh MP, Bhandari S, Nisar S, Poriya R (2022). Mass screening and treatment (MSaT) for identifying and treating asymptomatic cases of malaria-malaria elimination demonstration project (MEDP), Mandla, Madhya Pradesh. Malar J.

[22] Singh A, Singh MP, Bhandari S, Rajvanshi H, Nisar S, Telasey V (2022). Significance of nested PCR testing for the detection of low-density malaria infection amongst febrile patients from the malaria elimination demonstration project in Mandla, Madhya Pradesh, India. Malar J.

[23] Bharti PK, Alam MT, Boxer R, Shukla MM, Gautam SP, Sharma YD (2010). Therapeutic efficacy of chloroquine and sequence variation in pfcrt gene among patients with falciparum malaria in central India. Trop Med Int Health.

[24] Saha P, Guha SK, Das S, Mullick S, Ganguly S, Biswas A (2012). Comparative efficacies of artemisinin combination therapies in *Plasmodium falciparum* malaria and polymorphism of pfATPase6, pfcrt, pfdhfr, and pfdhps genes in tea gardens of Jalpaiguri District, India. Antimicrob Agents Chemother.

[25] Mishra N, Kaitholia K, Srivastava B, Shah NK, Narayan JP, Dev V (2014). Declining efficacy of artesunate plus sulphadoxine–pyrimethamine in northeastern India. Malar J.

[26] Mishra N, Singh JPN, Srivastava B, Arora U, Shah NK, Ghosh SK (2012). Monitoring antimalarial drug resistance in India via sentinel sites: outcomes and risk factors for treatment failure, 2009–2010. Bull World Health Organ.

[27] Mixson-Hayden T, Jain V, McCollum AM, Poe A, Nagpal AC, Dash AP (2010). Evidence of selective sweeps in genes conferring resistance to chloroquine and pyrimethamine in *Plasmodium falciparum* isolates in India. Antimicrob Agents Chemother.

[28] Sharma YD (2012). Molecular surveillance of drug-resistant malaria in India. Curr Sci.

[29] Sutar SKD, Dhangadamajhi G, Kar SK, Ranjit M (2013). Molecular monitoring of antimalarial drug resistance among *Plasmodium falciparum* field isolates from Odisha, India. Acta Trop.

[30] Sehgal PN (1998). Malaria: radical treatment for falciparum malaria in chloroquine resistant strain areas. Health Millions.

[31] Lumb V, Madan R, Das MK, Rawat V, Dev V, Khan W, Khan H, Sharma YD (2012). Differential genetic hitchhiking around mutant pfcrt alleles in the Indian *Plasmodium falciparum* population. J Antimicrob Chemother.

[32] Mallick PK, Joshi H, Valecha N, Sharma SK, Eapen A, Bhatt RM (2012). Mutant pfcrt SVMNT haplotype and wild type pfmdr1 N86 are endemic in *Plasmodium vivax* dominated areas of India under high chloroquine exposure. Malar J.

[33] Valecha N, Joshi H, Mallick PK, Sharma SK, Kumar A, Tyagi PK (2009). Low efficacy of chloroquine: time to switchover to artemisinin-based combination therapy for falciparum malaria in India. Acta Trop.

[34] Lynch C, Pearce R, Pota H, Cox J, Abeku TA, Rwakimari J (2008). Emergence of a dhfr mutation conferring high-level drug resistance in *Plasmodium falciparum* populations from southwest Uganda. J Infect Dis.

[35] McCollum AM, Basco LK, Tahar R, Udhayakumar V, Escalante AA (2008). Hitchhiking and selective sweeps of *Plasmodium falciparum* sulfadoxine and pyrimethamine resistance alleles in a population from central Africa. Antimicrob Agents Chemother.

[36] Pearce RJ, Pota H, Evehe M-SB, Bâ E-H, Mombo-Ngoma G, Malisa AL (2009). Multiple origins and regional dispersal of resistant dhps in African *Plasmodium falciparum* malaria. PLoS Med.

[37] Mishra S, Bharti PK, Shukla MM, Ali NA, Kashyotia SS, Kumar A, Dhariwal AC, Singh N (2017). Clinical and molecular monitoring of *Plasmodium falciparum* resistance to antimalarial drug (artesunate + sulphadoxine–pyrimethamine) in two highly malarious district of Madhya Pradesh, Central India from 2012–2014. Pathog Glob Health.

[38] Pathak A, Mårtensson A, Gawariker S, Sharma A, Diwan V, Purohit M (2020). Stable high frequencies of sulfadoxine–pyrimethamine resistance associated mutations and absence of K13 mutations in *Plasmodium falciparum* 3 and 4 years after the introduction of artesunate plus sulfadoxine–pyrimethamine in Ujjain, Madhya Pradesh, India. Malar J.

[39] Pathak A, Mårtensson A, Gawariker S, Mandliya J, Sharma A, Diwan V (2014). Characterization of drug resistance associated genetic polymorphisms among *Plasmodium falciparum* field isolates in Ujjain, Madhya Pradesh, India. Malar J.

[40] Sarmah NP, Sarma K, Bhattacharyya DR, Sultan AA, Bansal D, Singh N (2017). Antifolate drug resistance: novel mutations and haplotype distribution in dhps and dhfr from Northeast India. J Biosci.

[41] Kar NP, Chauhan K, Nanda N, Kumar A, Carlton JM, Das A (2016). Comparative assessment on the prevalence of mutations in the *Plasmodium falciparum* drug-resistant genes in two different ecotypes of Odisha state, India. Infect Genet Evol.

[42] Kumar A, Singh SP, Bhatt R, Singh V (2019). Genetic profiling of the *Plasmodium falciparum* parasite population in uncomplicated malaria from India. Malar J.

[43] Mohapatra PK, Sarma DK, Prakash A, Bora K, Ahmed MA, Sarma B (2014). Molecular evidence of increased resistance to anti-folate drugs in *Plasmodium falciparum* in North-East India. A signal for potential failure of artemisinin plus sulphadoxine–pyrimethamine combination therapy. PLoS ONE.

[44] Ashley EA, Dhorda M, Fairhurst RM, Amaratunga C, Lim P, Suon S (2014). Spread of artemisinin resistance in *Plasmodium falciparum* malaria. N Engl J Med.

[45] Ménard D, Khim N, Beghain J, Adegnika AA, Shafiul-Alam M, Amodu O (2016). A worldwide map of *Plasmodium falciparum* K13-propeller polymorphisms. N Engl J Med.

[46] Pacheco MA, Kadakia ER, Chaudhary Z, Perkins DJ, Kelley J, Ravishankar S (2019). Evolution and genetic diversity of the k13 gene associated with artemisinin delayed parasite clearance in *Plasmodium falciparum*. Antimicrob Agents Chemother.

[47] Chhibber-Goel J, Sharma A (2019). Profiles of Kelch mutations in *Plasmodium falciparum* across South Asia and their implications for tracking drug resistance. Int J Parasitol Drugs Drug Resist.

[48] Mishra N, Bharti RS, Mallick P, Singh OP, Srivastava B, Rana R (2016). Emerging polymorphisms in falciparum Kelch 13 gene in northeastern region of India. Malar J.

[49] Mishra N, Prajapati SK, Kaitholia K, Bharti RS, Srivastava B, Phookan S (2015). Surveillance of artemisinin resistance in *Plasmodium falciparum* in India using the kelch13 molecular marker. Antimicrob Agents Chemother.

[50] Das S, Saha B, Hati AK, Roy S (2018). Evidence of artemisinin-resistant *Plasmodium falciparum* malaria in eastern India. N Engl J Med.

[51] Das S, Manna S, Saha B, Hati AK, Roy S (2019). Novel pfkelch13 gene polymorphism associates with artemisinin resistance in eastern India. Clin Infect Dis.

[52] Bharti PK, Shukla MM, Ringwald P, Krishna S, Singh PP, Yadav A (2016). Therapeutic efficacy of artemether–lumefantrine for the treatment of uncomplicated *Plasmodium falciparum* malaria from three highly malarious states in India. Malar J.

[53] Duraisingh MT, Cowman AF (2005). Contribution of the pfmdr1 gene to antimalarial drug-resistance. Acta Trop.

